# Heat Shock Proteins: Connectors between Heart and Kidney

**DOI:** 10.3390/cells10081939

**Published:** 2021-07-30

**Authors:** Carolina Victória Cruz Junho, Carolina Amaral Bueno Azevedo, Regiane Stafim da Cunha, Ainhoa Rodriguez de Yurre, Emiliano Medei, Andréa Emilia Marques Stinghen, Marcela Sorelli Carneiro-Ramos

**Affiliations:** 1 Center of Natural and Human Sciences (CCNH), Laboratory of Cardiovascular Immunology, Federal University of ABC, Santo André 09210-580, Brazil; carolina.junho@gmail.com; 2 Experimental Nephrology Laboratory, Basic Pathology Department, Universidade Federal do Paraná, Curitiba 81531-980, Brazil; carolina.amaral1@ufpr.br (C.A.B.A.); regidacunha@gmail.com (R.S.d.C.); andreastinghen@ufpr.br (A.E.M.S.); 3 Laboratory of Cardioimmunology, Institute of Biophysics Carlos Chagas Filho, Federal University of Rio de Janeiro, Rio de Janeiro 21941-902, Brazil; ainhoardyg@gmail.com (A.R.d.Y.); emedei70@hotmail.com (E.M.); 4 D’Or Institute for Research and Education, Rio de Janeiro 21941-902, Brazil; 5 National Center for Structural Biology and Bioimaging, Federal University of Rio de Janeiro, Rio de Janeiro 22281-100, Brazil

**Keywords:** heat shock proteins, renal diseases, cardiac diseases, immune system, cardiorenal syndrome

## Abstract

Over the development of eukaryotic cells, intrinsic mechanisms have been developed in order to provide the ability to defend against aggressive agents. In this sense, a group of proteins plays a crucial role in controlling the production of several proteins, guaranteeing cell survival. The heat shock proteins (HSPs), are a family of proteins that have been linked to different cellular functions, being activated under conditions of cellular stress, not only imposed by thermal variation but also toxins, radiation, infectious agents, hypoxia, etc. Regarding pathological situations as seen in cardiorenal syndrome (CRS), HSPs have been shown to be important mediators involved in the control of gene transcription and intracellular signaling, in addition to be an important connector with the immune system. CRS is classified as acute or chronic and according to the first organ to suffer the injury, which can be the heart (CRS type 1 and type 2), kidneys (CRS type 3 and 4) or both (CRS type 5). In all types of CRS, the immune system, redox balance, mitochondrial dysfunction, and tissue remodeling have been the subject of numerous studies in the literature in order to elucidate mechanisms and propose new therapeutic strategies. In this sense, HSPs have been targeted by researchers as important connectors between kidney and heart. Thus, the present review has a focus to present the state of the art regarding the role of HSPs in the pathophysiology of cardiac and renal alterations, as well their role in the kidney–heart axis.

## 1. Heat Shock Proteins: Definition and Function

Heat shock proteins (HSPs) are a family of proteins produced by both unicellular and pluricellular organisms in response to different categories of stress conditions and were initially described by Ferruccio Ritossa in the early 1960s in *Drosophila melanogaster* [1,2]. However, it was not until the 1980s that these proteins were studied in depth by William Currie in heart tissue [3,4,5].

HSPs are stress proteins that possess molecular sizes ranging from 10 to 150 kDa, and they are found in all principal cellular compartments. These proteins were first discovered as cell protectors that function after exposure to high temperatures [6]. Researchers subsequently observed that HSPs also act as molecular chaperones that play critical roles in protein folding, intracellular protein trafficking, and the response to unfolded and denatured proteins resulting from heat and other stressors. Therefore, the study of HSPs has undergone explosive growth; however, their role in the context of cardiorenal syndrome (CRS) remains largely unexplored.

HSPs are proteins that are strongly conserved throughout the evolution of eukaryotes, and they protect organisms against injurious stimuli [7]. Normal levels of HSPs are required for the natural mechanism of protein folding, the maintenance of transduction signals, and development [8]. During pathological conditions, HSPs have been reported to respond to injuries caused by temperature, toxins, hypoxia, infectious agents, nitric oxide (NO), radiation, and other stressors. During these stimuli, HSPs are highly expressed [7]. 

In general, HSPs regulate the formation and trafficking of the protein complex, the refolding of mitochondrial and denatured proteins, the prevention and/or inhibition of protein unfolding and aggregation, and apoptosis [9]. In their anti-apoptotic role, these proteins regulate the activity of caspases, the c-Jun N-terminal kinase (JNK) pathway, and the nuclear factor kappa B (NF-kB) pathway [10]. In their anti-inflammatory role, HSPs suppress NF-kB, decrease pro-inflammatory cytokine levels, and/or stimulate damage-associated molecular patterns (DAMPs) in a manner that exacerbates them [10].

HSP families have been characterized according to their molecular weight. The most studied of these families are small HSPs (sHSPs), HSP60, HSP70, and HSP90. They can also be classified as stress-induced (when they are rapidly and highly expressed in response to stress) and those that function independent of stress (when they are constitutively expressed in cells) [11]. To perform their functions, HSP families possess singular structural domains and features. 

The sHSPs are a group of chaperones that are located predominantly in the cytosol and do not possess an ATPase domain. These proteins contain only a core α-crystallin domain (ACD) that is handed by variable N-terminal and C-terminal domains. They are considered small proteins of 12–43 kDa in size. In general, sHSPs function to stabilize injured proteins to prevent misfolded protein interactions and aberrant protein aggregation by exposing the hydrophobic residues of these proteins [12]. These types of HSPs operate prior to refolding attempts by the ATP-dependent complexes in a fixed order that includes flanking of the ACD, the use of polydispersive self-multimerization with other sHSPs, altering the subunit, and regulating the cell, and these processes can include a number of post-translational modifications [12]. In humans, 10 alpha-crystallin domains have been identified for the sHSPs, and these include HSPB1, HSPB2, HSPB3, HSPB4, HSPB5, HSPB6, HSPB7, HSPB8, HSPB9, and Hsbp10.

The HSP40/DNAJs family represents a heterogeneous group of co-chaperones characterized by the presence of the remarkably conserved J-domain, responsible for the regulation of the ATPase activity of HSP70s. DNAJs/HSP40s are chaperones by themselves as they bind to exposed hydrophobic residues of unfolded and nascent polypeptides and share common substrates with HSP70s [13,14,15].

HSP60 is comprised of a group of chaperones that are located predominantly in mitochondria and are organized as two curvy heptameric rings possessing three domains (apical, intermediate, and equatorial) that primarily function to assemble the mitochondrial protein folding apparatus [16]. HSP60s possess ATPase activity as evidenced by the ATP increase of the free energy of misfolded molecules. They interact with HSP10 to transport peptides from the cytosol to the mitochondria [17]. They possess a size of 60 kDa, and in addition to their classical HSP function, the HSP60s also are highly involved in the replication of mitochondrial DNA [18]. The involvement of extracellular HSP60 in inflammation activation processes has also been previously described. This type of HSP can act as a ligand for several receptors such as toll-like receptors (TLRs) and CD40 that trigger the release of inflammatory cytokines [19]. Previous studies have provided evidence that HSP60 can generate pro- and anti-inflammatory responses, thus corroborating the heat-shock paradox that will soon be further revised. HSP60 can also stimulate T cell activation and promote the functions of interferon gamma (IFN-γ), tumor necrosis factor alpha (TNF-α), NO, and IL-1, IL-6, IL-12, and IL-15 to generate a Th1-type response and T-cell adhesion to fibronectin through the TLR pathway [20].

The HSP70 group is characterized by a cytosol/nuclear molecule consisting of an N-terminal (44 kDa ATPase domain, ATP-dependent) that binds to and hydrolyzes the C-terminal substrate (28 kDa) domain associated with a short flexible linker of polypeptides [21]. The function of this group is associated with protein trafficking and degradation refolding of denatured proteins during stress, and these proteins also possess anti-apoptotic properties [22]. HSP70s appear to be important in the recognition of pathogen-associated molecular patterns (PAMPs) by the TLR4 pathway [23]; however, HSP70 is also considered to be a DAMP that activates the TLR cascade, as recombinant HSP70 has been previously observed to stimulate the synthase of pro-inflammatory cytokines (TNF-α, IL-1β, and IL-6) and NO by antigen presenting cells (APC) [22]. Finally, this HSP group is crucially involved in apoptosis. HSP70-1 binds to apoptosis protease-activating factor 1 (Apaf-1) and inhibits procaspase 9 in the apoptosome and caspase 3 [24,25]. HSP70 exhibits anti-apoptotic properties and can inhibit programmed cell death via intrinsic and extrinsic pathways. This protein is also involved in protein trafficking and degradation of denatured proteins under stress and possesses an ambiguous function regarding the immune system, where it can induce both pro-inflammatory and anti-inflammatory cytokines depending on where it is located [26,27]. HSP70 located in the cytosol appears to play a role in reducing pro-inflammatory pathways, while extracellular HSP70 (also referred to as a chaperokine) possesses strong immune-stimulatory effects [28]. This is in agreement with the “danger theory” described by Matzinger, where HSPs, particularly HSP70, released from damaged cells act as DAMPS and are recognized by APC [29]. APC is then activated to produce pro-inflammatory cytokines and stimulate NF-κB, thus initiating the adaptive immune response [30]. The HSP70 complexes with two co-chaperones, HSP40 and HSP110. HSP40 stimulates ATP hydrolysis, whereas HSP110 acts as a nucleotide exchange factor that accelerates ADP dissociation from the HSP70 [31,32].

The HSP90 family is characterized by a cytosolic homodimer with two isoforms (alfa and beta), where dimerization occurs at the C-terminus and nucleotide exchange occurs at the N-terminus [33]. These proteins are chaperones that interact with multiple charter proteins and function to adjust protein complex conformations such as those involved in RNA polymerase II, the telomere complex, the kinetochore, snoRNA, PI3K-related protein kinase (PIKK), the RNA-induced silencing complex (RISC), and the 26S proteasome [34]. HSP90 plays conflicting roles in cells and is essential for the maintenance of metabolism and for cell apoptosis. Its normal role involves the maintenance of healthy cells; however, during its dysregulation in cancer cells, HSP90 may assist the carcinogenesis. That is the reason HSP90 inhibitors are used to treat certain types of cancer, thus suggesting its value as a therapeutic target [35].

The HSP110 family act as nucleotide exchange factors for both HSP70 proteins, as mentioned. They are also known as highly capable to hold (unfolded) proteins in a folding competent, acting as coupling factors between the nucleotides [36]. 

As described above, HSPs can play double roles during pathological situations and can act as chaperones and anti-apoptotic modulators in response to pro-inflammatory and pro-oxidative stress. It has been demonstrated that intracellular and extracellular HSPs possess distinct functions during injury [26]. While the extracellular HSPs are described as agonists for TLRs and DAMPS, intracellular HSPs appear to decrease inflammation and inhibit the release of reactive oxygen species (ROS) [7]. After several years of studies focused on deciphering the active mechanisms underlying this paradox, Demeester et al. revealed that when the HSP causes inflammation, cytoprotection is being promoted, and when inflammation activates the HSP, cell death is promoted [30]. Twenty years after the proposal of this paradox, only a small number of studies have attempted to further elucidate it. The majority of the current explanations for this paradox involve the participation of NF-κB and how this nuclear factor behaves during pathological conditions [7].

Cardiorenal involvement of HSPs as its own subspecialty remains at a very early stage. In the literature, the implications of HSPs in heart and kidney pathologies have previously been separately characterized, thus reinforcing the dual role of intra-and extracellular HSPs in the emergence and aggravation of these diseases (Table 1).

## 2. Participation of HSPs in Cardiorenal Diseases

### 2.1. HSPs and Heart

Currie, in agreement with the initial discoveries in *Drosophila*, demonstrated that in the heart, metabolic stress was also able to induce higher “SP71” synthesis [37]. Furthermore, the expression of HSPs is enhanced not only by metabolic insults [38] but also by other types of stress such as ischemia at the heart level [39]. Thus, as Dillmann et al. observed in the ischemic area of dog hearts after occlusion of the left anterior descending coronary artery [40], Currie in perfused rat hearts demonstrated that HSP70 acts as an indicator of cellular stress, thus indicating that not only ischemia but also perfusion at supra-optimal temperature increases the synthesis of this protein as a typical heat-shock response [5]. In the heart, HSP70 prevents disease and protects cardiomyocytes from stress [41]. The expression of HSP70 is elevated in myocardial tissues following cardiac surgery, general surgery, or ischemia [42]. Studies have reported a diminished incidence of postoperative atrial fibrillation (AF) in patients with high levels of intracellular HSP70, and these findings are contrary to those in patients with low HSP70 who exhibit an increased risk of postoperative AF [43,44,45].

Hyperthermic treatment followed by a prolonged recovery period was demonstrated not only in vitro but also in vivo (as opposed to the case where there is no accumulation of SP71) to suppress the synthesis of SP71 as a protective method [4,5]. These studies highlighted the possibility of an alternative therapy to reduce the ischemic heart area during reperfusion in heart transplantation procedures [46,47].

To further examine the origin of the synthesis of HSP70, Dybdahl et al. conducted a study involving patients who underwent coronary artery bypass grafting, a procedure that is known to cause an inflammatory response due to stress and to mobilize HSP70 proteins in response to that stress. The study executed by Dybdahl et al. revealed that TLR2 and TLR4 play essential roles in the HSP70 signaling pathways linking this protein to the immune response, thus highlighting the role of HSPs as tissue damage markers [48]. In addition, HSP70 levels were increased in both coronary heart diseases [49] and of hypertension-induced cardiac hypertrophy and fibrosis [50]. Moreover, Cai et al. differentiated between the role of intracellular and extracellular HSPs, as they exhibit differentiated functions where extracellular HSPs are more relevant in protection against ischemia and metabolic stress [50].

Several works also explored the role of HSP70 in genetically manipulated mice. Thus, Hutter el at. demonstrated that HSP72 overexpression reduced infarct size in this in vivo transgenic mouse model of myocardial ischemia and reperfusion [51]. In the same line, Radford et al. generated lines of transgenic mice to express human 70-kDa heat shock protein constitutively in the myocardium demonstrating a direct cardioprotective effect of 70-kDa heat shock protein to enhance postischemic recovery of the intact heart [52]. Importantly, using HSP70 overexpression transgenic mice, Naka et al. were able to shown the cardioprotective effect of this protein against cardiac dysfunction induced by doxorubicin [53]. Conversely with these data, Bernardo et al. showed that the sustained overexpression of HSP70 was not enough to prevent cardiac dysfunction, conduction abnormalities, fibrosis or characteristic molecular markers of the failing heart. However, the authors suggested that this overexpression could give protection in acute cardiac stress settings, but appears insufficient to protect the heart under chronic cardiac disease conditions [54].

As HSP40 appears as a co-chaperone of HSP70, a crucial role of mitochondrial Hsp40 in preventing dilated cardiomyopathy. Hayashi et al. described the participation of HSP as crucial for mitochondrial biogenesis once it was found that interacting proteins identified the alpha-subunit of DNA polymerase gamma (Polga) as a client protein [15].

The extracellular HSPs in cardiac tissue can function via TLR4 activation to stimulate pro-inflammatory cytokines, and therefore, the role of HSPs in cardiac function preservation can overcome TLR4 pathway suppression [55]. In this regard, the authors consistently demonstrated that HSP70 preconditioning attenuates cardiac TNF-α, NF-κB, and intercellular adhesion molecule 1 (ICAM-1) levels. Interestingly, the authors also revealed that the TNF-α response to endotoxin is mitigated by HSP70 preconditioning in macrophages in vitro.

Due to the role of HSPs in cardiac function, these molecules have also been identified as important biomarkers. Evidence supporting this was provided by studies related to acute coronary syndrome (ACS). Zhang et al. demonstrated that there is a close relationship between the presence of HSP70 in plasma and the risk of developing cardiac disturbances. Moreover, high levels of HSP70 and low levels of anti HSP70 within plasma are associated with a higher risk of cardiac disturbances and a higher severity of these disturbances [56].

In regard to atrial fibrillation (AF), it was observed that elevated levels of serum HSPs (HSP70 or HSP27) were indicators of higher AF recurrence after ablative therapy [57,58]. This highlights the relevance of these proteins as biomarkers, as the overexpression of HSPs after heart damage reflects a protective effect [59]. It is important to note that van Marion et al. did not observe an association between basal levels of HSP and AF during the presence or recurrence of heart disease. The authors observed that patients with AF presented an increase in HSP27 serum levels within one year after pulmonary vein isolation, thus suggesting that HSP27 levels may predict the recrudescence of AF after ablative therapy [58]. In agreement these results, Hu et al. revealed that HSP27 serum levels were related to left atrial voltage, left atrial diameter, and fractionated intervals, and they could predict AF relapse after catheter excision [60]

HSP110 also seems to be present during myocardial remodeling. Mohamed et al. showed that KO animals and cells for HSPA4 (HSP110 family member) impaired chaperone activities and increased cardiomyocyte area, expression of hypertrophy genes and contraction [31]. 

Krenek et al. demonstrated increased HSP90 expression in failing left ventricles in vivo [61]. However, HSP90 may play a cytoprotective role in cardiac injuries induced by high glucose and hypoxic postconditioning [62,63]. This suggests that the type of injury influences the cellular mechanisms of HSP. Additionally, serum HSP90 levels are also elevated in patients with carotid atherosclerosis [64] and HSP90 has been demonstrated to be overexpressed in human atherosclerotic plaques [64,65]. Studies have revealed that HSP90 participates in cardiac remodeling by inducing hypertrophy and collagen deposition, both of which are processes that compromise cardiac function and are related to heart failure [66,67,68]. In the last decade, pharmacological interventions have emerged to modulate the expression and function of HSPs to improve cardiac function. Studies examining not only intracellular levels of HSPs but also extracellular HSPs levels have revealed new insights into new paths for the diagnosis of heart failure and possible therapy [69]. Thus, inhibition/modulation of these HSPs has emerged as a potential therapeutic target. In this regard, Yoon et al. demonstrated that HSP inhibition by 2-Phenylethane sulfonamide attenuated cardiac hypertrophy induced by aortic banding and phenylephrine in neonatal ventricular cardiomyocytes in mice [70]. In agreement with this, Marunouchi et al. recently demonstrated that therapeutic effects were promoted by the inhibition of HSP90 by minimizing the stimulation of the RIP1-RIP3-MLKL pathway in cardiac hypertrophy [71]. In addition, Liu et al. used a doxorubicin-induced left ventricular dilation and heart dysfunction model to demonstrate that blocking HSP70 activity with antibodies significantly ameliorated cardiac heart function [72].

### 2.2. Cardiorenal Syndrome

The kidneys and heart are essential organs that are required for proper functioning of the body. The function of the heart is associated with pumping blood throughout the body, while the kidneys clean the blood, remove toxins and excess metabolites, and control blood pressure. Although these appear to be easy assignments, they demand delicate and accurate processes that are dependent upon each other. Since the 1830s, the connection between the heart and kidneys has been studied. The first study examining this connection was published in 1836 by Robert Bright after he observed the prevalence of cardiovascular diseases (CVD) in patients with renal disease that were accompanied by the secretion of urinary albumin [73].

After this initial observation, studies examining the connection between the heart and kidneys revealed a specific disorder termed cardiorenal syndrome (CRS). In 2008, the Acute Dialysis Quality Initiative defined the newest description and classifications of CRS that included two major CRS groups (cardiorenal and reno-cardiac) based on the initial pathology. These are further sub-grouped into five types of CRS [74]. Based on this, CRS is defined by meaningful heart–kidney connections that divide similarities in pathophysiology, where an injury in one of the organs leads to an injury in the other.

Both type 1 and type 2 are considered cardio-renal where the primary injury occurs in the heart. Known as CRS type 1, acute cardio-renal syndrome is initially defined by acute loss of cardiac function leading to acute kidney injury (AKI) that occurs primarily through hemodynamic mechanisms. Termed CRS type 2, chronic cardio-renal syndrome is defined as chronic cardiac diseases leading to chronic kidney disease (CKD). Chronic renal congestion caused by cardiac events increases the pressure, and this increases the risk of CKD [75].

Types 3 and 4 are considered reno-cardiac, where the primary injury occurs in the kidneys. Termed CRS type 3, acute reno-cardiac syndrome is defined as AKI and causes acute heart injury. Termed CRS type 4, chronic reno-cardiac syndrome is defined by a cardiovascular impairment in patients affected by CKD. Finally, type 5 CRS is defined as systemic diseases such as sepsis, hepatorenal syndrome, diabetes, and diseases related to the immune system that can induce cardiac and renal dysfunction simultaneously [76,77].

The study of CRS is of paramount importance to develop effective clinical treatments, as heart problems represent the largest cause of death in the world at approximately 15 million deaths per year [78]. According to the World Health Organization (WHO), approximately 43% of deaths caused by CVD were in patients who exhibited some level of kidney failure, thus clinically evidencing the link between the heart and kidneys. The inflammatory process covers all CRS types as a type of starting point. Cytokine release appears to be the primary cardiorenal connector, as cytokines can interact directly with heart tissue. It is established that blood tumor necrosis factor-alpha (TNF-α), interleukin (IL-) 1, and IL-6 are all increased during CRS [79,80]. This inflammatory process can be initiated or maintained by HSPs, the essential proteins involved in the control of gene transcription and intracellular signaling in addition to acting as important modulator of the immune system [6].

### 2.3. Role of HSPs in Cardiorenal Syndrome

HSP90 proteins critically involved in the modulation of several cell signaling pathways; however, the expression and even the functions of HSP90 may be altered under pathological conditions [81]. Indeed, HSP90 expression is affected by indoxyl sulfate, a uremic toxin that accumulates in the body during CKD progression. Milanesi et al. demonstrated that indoxyl sulfate induces HSP90 expression in kidney fibroblasts (NRK-49F cells) [82]. However, in these cells selective HSP90 inhibition reverses the inductive effect of indoxyl sulfate on monocyte chemoattractant protein-1 (MCP-1), α-smooth muscle actin, collagen I, and transforming growth factor-β (TGF-β) expression, thus indicating that HSP90 contributes to kidney inflammation and fibrosis at the cellular level [72]. In vivo, the authors also observed an increase in HSP90 expression in the kidneys of mice treated with indoxyl sulfate [82]. Furthermore, clinical studies have demonstrated that the HSP90α isoform is present at elevated serum levels in pediatric patients with CKD compared to levels in the control group [83].

HSP90α interacts directly with endothelial nitric oxide synthase (eNOS), and this interaction leads to enhanced enzyme activity and subsequently to increased production of NO, an important mediator in endothelium-dependent vasodilation. Interestingly, Amador-Martinez et al. demonstrated a decrease in the interaction between HSP90α and eNOS occurred in the hearts of CKD model rats [84]. This could contribute to the reduction of NO bioavailability, which is a marker of endothelial dysfunction. These findings suggest that renal damage in CKD induces changes in HSP90 function in the cardiovascular system in the context of a CRS type 4 model. In another study, Barrera-Chimal et al. demonstrated that HSP90 and eNOS interactions are also impaired in IRI in AKI model rats [85]. The authors demonstrated that animals subjected to IRI exhibited a decrease in renal blood flow and NO formation as assessed by a significant reduction in urinary nitrite and nitrate excretion; however, these effects were attenuated by intra-renal transfection of HSP90α and HSP90β isoforms [85]. Moreover, HSP90 inhibition using radicicol exerts a negative impact on GFR and renal blood flow in animal models [83]. Therefore, these data indicate that HSP90 contributes to the NO/eNOS pathway and to the regulation of renal vascular tone [85,86].

Notably, HSP90 inhibition reduces the inflammatory response, pro-oxidative factors, and the migration and proliferation of vascular smooth muscle cells (VSMC) in atherosclerosis [65,87]. In this regard, a number of studies have also shown that HSP90 inhibition may play a beneficial role in cardiovascular disorders triggered by other diseases. Lazaro et al. demonstrated that HSP90 inhibition reduces atherosclerotic lesions and renal damage in a diabetic mouse model [88]. These data suggest that HSP90 may improve vascular injury and also as well as nephropathy in diabetes that is strongly linked to cardiovascular and kidney dysfunction and is associated with CRS development [89,90].

In a study examining IRI, Zhang et al. demonstrated an increased renal expression of HSPs, particularly of HSP70 and HSP27, and these were identified as 43-fold and 12-fold increases, respectively [49]. The evidence of a cytoprotective function of HSPs after IRI was reinforced by a study by Wang et al. that reported a decrease in kidney function, tubular injury, and survival ratio in HSP70^−/−^ mice [91]. These results indicate that HSP70 can promote renal epithelial cell survival and preserve organ function after ischemia, and this is achieved in part by modulating AKT and glycogen synthase kinase 3-β (GSK3β) activity [91]. The precise mechanism by which HSP70 reduces injury remains unclear. However, it has been hypothesized that this may result from the prevention of NF-κB p65 translocation or IkB stabilization [92,93,94,95].

In vitro studies revealed increased HSP72 (a member of the HSP70 family) levels that appear to exert a crucial cytoprotective effect under elevated urea levels [96,97]. In contrast, a previous study observed decreased levels of HSP72 in blood monocytes of patients with CKD in pre dialysis, while unchanged serum levels of HSP70 were detected in children with CKD [83,98]. Lebherz-Eichinger et al. demonstrated that CKD patients at stage 4 and 5 exhibited elevated HSP70 urinary values and increased fractional HSP70 excretion in stage 5 when compared to healthy controls, while most serum samples exhibited values that were below the threshold [99]. HSP70 in urine may be derived from renal cells as a response to augmentation of uremic stress and could be quickly excreted, and this explains, at least in part, why the serum levels remain unaffected [99].

Regarding patients undergoing dialysis treatment, studies examining peripheral blood monocytes demonstrated that HSP72 mRNA levels were significantly lower in adults undergoing hemodialysis (HD) than they were in controls. Interestingly, when urea-treated macrophages harvested from healthy controls were exposed to heat stress, a significant increase in the expression of HSP72 was observed immediately after incubation with urea alone. These findings suggested that although the stress response was altered due to CKD, the stress response was not fully abrogated [100]. Aufricht et al. revealed in vitro that mesothelial cells exposed to commercial peritoneal dialysis fluid (PDF) exhibited a rapid accumulation of HSP70 [101]. The same group demonstrated in 2003 [99] that in vivo exposure of mesothelial cells to PDF induces HSP72 overexpression [102]. Bender et al. used pharmacological manipulation of the peritoneal dialysis solution (PDS) through the addition of glutamine to improve the status of mesothelial cells in vitro by inducing the expression of HSP27 and HSP72 [99]. In an in vivo model, the addition of glutamine also decreased the amount of protein that was lost in PDS [103].

Lu et al. demonstrated that HSP72 induction can prevent the development of vascular calcification in human aortic smooth muscle cells [104]. Vascular calcification is correlated with cardiovascular mortality and is commonly observed in patients with coronary artery disease and CKD initiated by CRS type 4 [105,106]. Clinical studies have also revealed a reverse relationship between circulating HSP72 and the presence of coronary artery disease and the degree of atherosclerosis [107].

In regard to the cardiorenal connection itself, few studies have examined the interaction of HSPs in a CRS model. One of these studies was by Trentin-Sonoda et al. that incorporated the use of a model of renal IRI that was analyzed by qPCR to assess the gene expression of HSP60 and 70 in the heart tissue of wild type and TLR2 and TLR4^−/−^ mice. In wild-type mice, there was an increase in the expression of these proteins that was not observed in the knockout mice, thus supporting the idea that the TLRs and HSP60/70 interact in the pathway that culminate in renal IRI-induced cardiac hypertrophy and dysfunction [80].

As mentioned previously, HSP27 belongs to the family of sHSPs and is a relevant inhibitor of the apoptotic intracellular pathway, as it can interact with pro-apoptotic components such as the caspase pathway [108]. HSP27 exerts antioxidant activity to lower ROS levels by increasing the intracellular levels of glutathione and by decreasing intracellular iron [109]. Additionally, HSP27 may contribute to decreased low-density lipoprotein (LDL) oxidative modification due to reduced ROS formation and also to its ability to compete with oxLDL uptake by macrophages, thus demonstrating that HSP27 plays a protective role in atherogenesis [110,111,112]. Keezer et al. demonstrated that HSP27 can avoid endothelial cell proliferation and migration and also migration stimulated via endostatin and thrombospondin-1 [113]. Furthermore, HSP27 promotes the production of anti-inflammatory cytokines by monocytes and inhibits TLR4 expression and differentiation into dendritic cells [114].

In a study examining HSP27 expression in human atherosclerotic plaques in patients with ACS, Park et al. observed a significant augmentation of HSP27 expression in adjacent normal-appearing vessel areas compared to that in control vessels [115]. De Souza et al. analyzed cardiac biopsies from patients who underwent heart transplantation and observed a 20-fold increase in the expression of HSP27 in patients who did not develop cardiac allograft vasculopathy (CAV) compared to patients who had developed CAV, thus suggesting an association of HSP27 with freedom from CAV [116]. Additionally, HSP27 expression is altered in CVD-like congestive heart failure [117]. A number of studies have shown that HSP27 is overexpressed in cardiac myocytes following ischemia-reperfusion [118,119,120]. Additionally, enhanced HSP27 levels have been reported to exert substantial functions in cardioprotection [121]. In this regard, HSP27 exhibits cardioprotective action through its anti-apoptotic and antioxidant properties and maintenance of the integrity of microtubules and actin cytoskeleton, and it also attenuates atherogenesis after modifying inflammation within the plaque. HSP27 can also modulate lipid uptake and possesses the ability to protect the ischemic endothelium [122].

HSP27 is detected in the endothelium of normal human kidneys as well as the distal tubules and collecting ducts [123]. Guo et al. observed increased HSP27 expression after acute ischemic kidney damage in rats [124]. As mentioned, Lebherz-Eichinger et al. performed a study examining patients with CKD and revealed increased serum levels of HSP27 in patients in stages 3 and 5 compared to the levels in healthy controls [99]. A high concentration of urinary HSP27 was observed in CKD stages 2 and 5. Positive correlations for age, C-reactive protein (CRP), and HSP27 serum concentrations were identified in these patients. In contrast, a negative correlation between eGRF and HSP27 serum levels was observed. The authors suggest that the enhanced HSP27 urine levels may be a result of renal compensatory activity due to increased HSP27 serum levels and kidney damage [99]. Jaroszyński et al. demonstrated that lower serum levels of HSP27 in patients on HD are related to carotid atherosclerosis and oxidative stress, and they also revealed that HSP27 is independently associated with sudden cardiac death (SCD) in patients undergoing HD treatment. Based on these results, it is likely that HSP27 acts as one of the many linking molecules influencing cardiovascular mortality in HD patients [110].

A study concerning glomerular diseases observed that the HSP40 (DNAJB9) protein was one of the most abundant proteins after proteomics analysis [14]. The group set this protein as a 100% sensitivity and 100% specificity biomarker of fibrillary glomerulonephritis. Glomerulonephritis is one common renal chronic disease that is already studied to cause cardiac injuries such as hypertension, heart failure, pulmonary edema, and damage to other organs as well as the increase of inflammatory cytokines [125], referring to CRS type 4 [75].

HSP60, also known as HSPD60 or HSPD1, acts as an important chaperone for mitochondrial protein folding and also modulates apoptosis [126]. However, under stress conditions HSP60 can be translocated to the cytosolic compartment, transported to the cell surface, and released from the cell [127,128]. In the extracellular space, HSP60 is immunogenic and can act as a signal for the immune system [126,127,129]; in patients with acute myocardial infarction, coronary heart disease, and carotid atherosclerosis, the serum levels of HSP60 are elevated [130,131].

According to another study, serum levels of HSP60 were associated with the risk of death and readmission in patients with acute heart failure [132]. In an experimental model, it was demonstrated that extracellular HSP60 induces apoptosis in cardiac myocytes via TLR4 [133]. A previous study from our group observed the involvement of HSP60 in cardiomyocyte hypertrophy and its association with inflammation and TLR4 activation. In this study, primary culture of cardiomyocytes treated with HSP60 showed hypertrophy, increase on complement system components, C3 and factor B as well as an increase in IL-6 and TNF-α expression [134].

It is also established that cardiac myocytes can release HSP60 in exosomes; however, the role of HSP60 in intercellular communication through extracellular vesicles remains unclear [135]. Additionally, studies have demonstrated that atherosclerotic lesions exhibit increased HSP60 expression [136]. HSP60 can also regulate important cellular mechanisms such as VSMC migration and proliferation that could contribute to atherosclerosis and endothelial damage [137].

Despite its importance in the cardiovascular system, few studies have investigated the role of HSP60 in kidney diseases. Fang et al. demonstrated that HSP60 is a target of miR-382 that reduces its expression in renal cells and contributes, at least in part, to renal tubulointerstitial fibrosis that is related to CKD progression [138]. In diabetic nephropathy, HSP60 may also be involved in renal tubular cell dysfunction [139,140].

**Figure 1:**
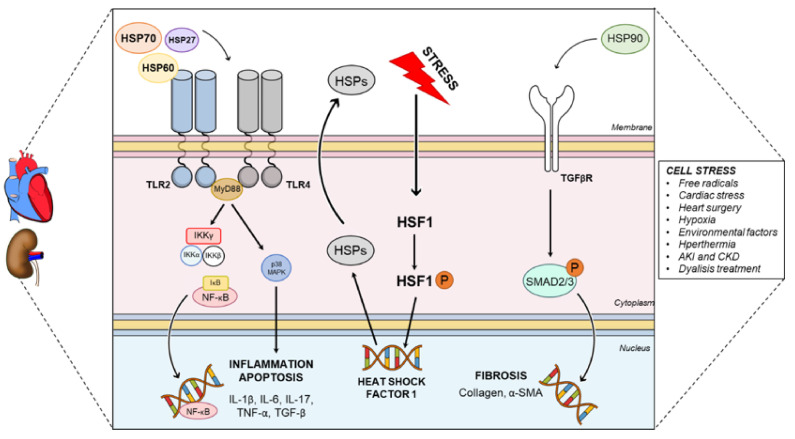
Representative figure showing the general intracellular mechanisms of action of HSPs in heart and kidney. Normally in the cardiorenal axis HSPs 27, 60 and 70 are the ligands of TLR2 and TLR4 (more found in the hearts and kidneys), activating the p38 and IKK-NF-kB pathways. This pathway is responsible for inducing the expression of inflammatory and apoptosis genes (IL-1β, IL-6, IL-17, TNF-α and TGF-β). On the other side, the HSP90 is responsible for activating TGF-βR/SMAD2/3 pathway, responsible for inducing the expression of fibrotic genes (α-SMA and collagen). Physiological cell stress can also induce the production of endogenous HSPs. They are required to access to HSF1 complex present in the cytosol, allowing for its phosphorylation (P). The phosphorylated complex enters the nucleus and increases HSP expression in the cellular cytosol and outside. IL: interleukin; TLR: toll-like receptor, TNF-α: tumor necrosis factor alfa; TGF: transforming growth factor; TGFR: transforming growth factor receptor; HSF1: heat shock factor 1; SMA: smooth muscle actin.

In summary, the most powerful system of myocardial-renal HSP-targeted is represented in the Figure 1. In general, the TLR2/4 pathway is the one that interacts with both organs and innate immune system. This conversation between heart–kidneys axis and HSPs depends on different molecular mechanisms of action. In general, HSP27, 60 or 70 couple to TLRs activating IKKγ or MAPK/p38 pathways. In the nucleus, NF-kB is responsible for the inflammatory gene expression while p38 activates the apoptotic genes [27,141]. This inflammatory response is observed in many cardiac injuries and kidney diseases, in addition of CRS itself [80]. Not only can this inflammation be induced by HSPs in CRS, but also the fibrosis observed during the syndrome [41,142]. The interaction of HSP90 with the receptor of TGF-β has been described to stimulate fibrosis by SMAD2/3 in renal tissue and can also promote fibroses in heart [143]. Last, but no less important, stress factors (free radicals, hypoxia, environmental factors, etc.) caused by CRS can directly induce an increase on HSPs expression by HSF1 phosphorylation. The activation of HSF1 has already been studied to cause cardiac dysfunction [144] and cause more apoptosis during renal injury [145].

**Figure 2:**
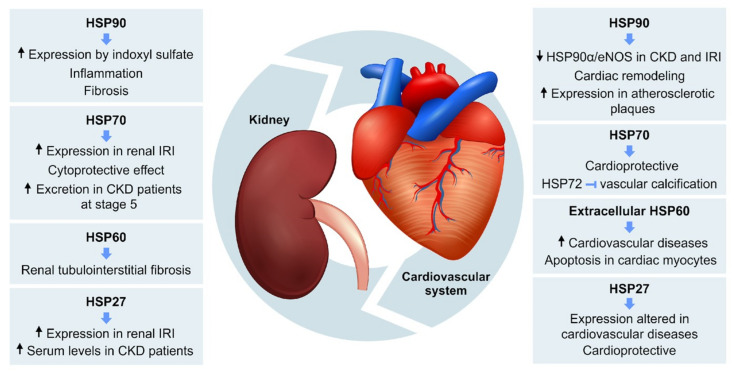
Schematic model showing the main role of HSPs in renal and cardiovascular diseases. HSP90, HSP70, HSP60 and HSP27 participate in pathophysiological mechanisms of renal and cardiovascular disorders, including the relationship between them in cardiorenal syndrome. HSP: heat shock proteins; CKD: chronic kidney disease; IRI: ischemia and reperfusion injury; eNOS: endothelial nitric oxide synthase.

Therefore, the cardiovascular and renal systems are strongly linked and present a complex relationship in which HSPs may be relevant in the pathological processes that affect these two systems (Figure 2). However, our knowledge regarding the role of HSPs in CRS is incomplete, and further studies are required. Based on the complexity of the relationship between cardiovascular and renal diseases, understanding the pathophysiological mechanisms involved in this process, including the possible role of HSPs, may be relevant for the development of new therapeutic strategies for CRS. 

## 3. Final Considerations

HSPs are known to participate in normal cellular function such as their well-known role in protein folding. However, the roles of HSPs in the context of different pathological processes remain to be fully elucidated, and studies are currently underway to further the understanding of their cellular mechanisms to potentially aid in the development of promising new strategies for diagnosis and treatment.

In regard to cardiovascular and kidney diseases, the literature has focused extensively on the role of HSPs in the onset and maintenance of pathological states. The ability of HSPs to control gene transcription after different types of injury such as cardiac ischemic processes or AKI is currently under rigorous study by the scientific community. Furthermore, inflammatory pathologies such as nephropathies and atherosclerosis have exhibited an important correlation between the levels of HSPs and the mortality rate observed in these diseases. It is important to highlight that inflammatory processes and, consequently, immune responses are modulated by HSPs, thus identifying them as complex molecules possessing a broad spectrum of action.

In this sense, the possibility of applying pharmacological agents to induce or inhibit the expression of HSPs as a means of preventing kidney and heart diseases, and even in organ transplantation, has been demonstrated to be important according to translational studies. Despite the great advances in knowledge regarding the role of HSPs in cellular processes, we are still far from understanding the entire process. Thus, a better understanding of how HSPs mediate kidney–-heart axis disease is required.

## Figures and Tables

**Table 1:** Correlation between different types of CRS and HSP-mediated cardiovascular diseases.

Classification	CRS Types	Pathologies	HSPs
Cardio-renal	1	Decompensated heart failure, congestion, acute coronary injury, acute kidney injury	HSP70
	2	Chronic heart failure, coronary heart disease, chronic kidney disease	HSP60 HSP90
Reno-cardiac	3	Renal ischemia, acute renal failure, arrhythmia, acute heart failure, myocardial infarction, atrial fibrillation, cardiac hypertrophy	HSP27 HSP60 HSP90
	4	Chronic kidney disease, uremic toxins accumulation, diastolic dysfunction, myocardial remodeling	HSP70 HSP72 HSP90
Systemic	5	Sepsis, cirrhosis, diabetes	HSP60 HSP90

## Data Availability

Not applicable.

## Abbreviations

ACD	α-crystallin domain
ACS	acute coronary syndrome
ADHF	acute decompensated heart failure
AF	atrial fibrillation
AKI	acute kidney injury
Apaf-1	apoptosis protease-activating factor 1
APC	antigen presenting cell
CAV	cardiac allograft vasculopathy
CKD	chronic kidney disease
CRS	cardiorenal syndrome
CVD	cardiovascular diseases
DAMPs	damage-associated molecular patterns
eNOS	endothelial nitric oxide synthase
ESKD	end-stage kidney disease
GFR	glomerular filtration rate
GSK3β	glycogen synthase kinase 3-β
HD	hemodialysis
HSPs	Heat Shock Proteins
ICAM-1	Intercellular adhesion molecule 1
IL	interleukin
IRI	ischemia and reperfusion injury
JNK	c-Jun *N*-terminal kinase
LDL	low-density lipoproteins
MCP-1	monocyte chemoattractant protein-1
MKBP	myotonic dystrophy kinase-binding protein
NF-kB	nuclear factor kappa B
NO	nitric oxide
PAMPs	pathogen-associated molecular patterns
PDF	peritoneal dialysis fluid
PDS	peritoneal dialysis solution
PIKK	PI3K-related protein kinase
RISC	RNA-induced silencing complex
ROS	reactive oxygen species
SCD	sudden cardiac death
sHSPs	small HSPs
TGF-β	transforming growth factor-β
TLRs	toll-like receptors
TNF-α	tumor necrosis factor-alpha
VSCM	vascular smooth muscle cells
WHO	World Health Organization
